# Unexpected adverse event of insulin therapy in diabetes mellitus

**DOI:** 10.1007/s00592-021-01674-1

**Published:** 2021-01-25

**Authors:** Matteo Borro, Giuseppe Murdaca, Mario Monachesi, Simone Negrini

**Affiliations:** 1 Department of Internal Medicine (DiMI), Clinical Immunology Unit, University of Genoa and Policlinico San Martino, Viale Benedetto XV 6, 16132 Genova, Italy; 2 Department of Internal Medicine, San Paolo Hospital, Via Genova 30, 17100 Savona, Italy; 3 SSD Endocrinologia E Diabetologia, ASL2 Savonese, Via Manzoni 14, 17100 Savona, Italy

**Keywords:** Type 2 diabetes mellitus, Insulin therapy, Pen needle, Adverse event

## Background

Type 2 diabetes mellitus is a metabolic disorder whose main finding is chronic hyperglycemia due to a mix of peripheral insulin resistance and secretory insulin deficiency. Duration and severity of abnormal glycemia affect patient outcome with cardiovascular complications being responsible for about 50% of mortality. Even microvascular complications, including neuropathy, retinopathy, and nephropathy, present an increased incidence correlated to duration of diabetes. In particular, the prevalence of neuropathy significantly increases and correlates with the progression from normal glucose tolerance (7, 4%) to overt diabetes (28%) [1]. The symmetric sensorimotor polyneuropathy is the most common form of diabetes-related neuropathy caused by metabolic and micro-vessel alterations as a result of chronic hyperglycemia exposure [1]. Treatment of chronic hyperglycemia should be started as soon as possible comprehending insulin therapy (IT) when indicated. Delaying the introduction of IT may impact on chronic complications [2]. Insulin pens have become the most common injection devices, and the 4-mm needle is now recommended for adults and children regardless of age, sex, ethnicity, or BMI. It has demonstrated to be safer, better tolerated, less painful and equivalently effective than the 8-mm needle that showed an higher risk of intramuscular injection [3]. The correct use of insulin devices comprehends the proper knowledge of the specific device, the verification of free and unobstructed flow before injection, the adequate cleaning, spacing and rotation between sites of injection, the appropriate time and the exact direction to push the thumb button, such as vertically, and the duration of the pressure on it until the needle is completely withdrawn from the body [3]. Moreover, after injection, needles should be disposed of with appropriate training. In fact, pens present with two different caps: an inner cap that protects the patient from the needle and the external cap that should be recapped before the safe removal of the needle itself. Unfortunately, compliance to devices is suboptimal and adverse events are relatively frequent [4].

## Objective

Here, we report a peculiar case report of an adverse events related to insulin pen use in a type 2 diabetes mellitus patient.

## Case report

**Fig. 1:**
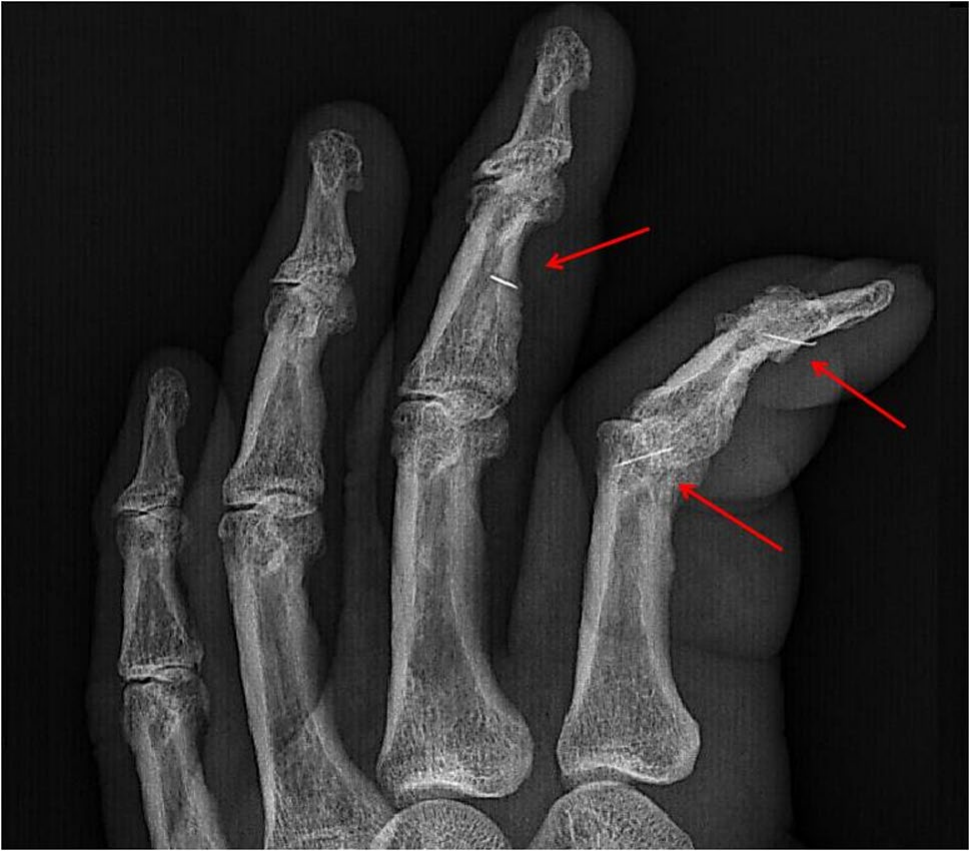
Enlargement of the oblique image of the left hand showing the three foreign bodies (red arrows) and the evident inflammatory edema of second and third fingers (color figure online)

A 68-year-old male presented to the Emergency Department with complaints of pain, swelling and functional limitation of the second and third fingers of the left hand. Medical history included insulin-treated type 2 diabetes mellitus complicated by severe peripheral polyneuropathy and chronic kidney disease. On physical examination, there were signs of cellulitis extending from the metacarpophalangeal joints to the distal phalanges of the second and third left fingers. Patient denied any trauma or injury to the site. Hand X-rays were performed and revealed three different foreign bodies compatible with metal splinter or needles: two were located in the second finger, close to the last interphalangeal joint and in the distal epiphysis of first phalanx respectively, and one was detected in the diaphysis of the second phalanx of the third finger (Fig. 1). After a course of systemic non-steroidal anti-inflammatory drugs and amoxicillin/clavulanic acid 875/125 mg / 8 h, foreign bodies were surgically removed and have been identified as 4-mm insulin pen needles. On questioning, because of its severe sensitivity loss, patient revealed an extreme difficulty in correctly recapping the needle of insulin pen with the external protection before safely discharge the device. He then confirmed that, holding the pen with his left hand, he sometimes broke the needle while recapping the pen. Sensitivity loss due to diabetic polyneuropathy prevented the patient from feeling pain when needles broke into his fingers and only the signs of inflammation revealed their presence. Accordingly, patient was instructed on the proper technique to safely discharge the needle and recap the device after injections.

## Discussion

The recommended insulin injection sites are the abdomen, thigh, buttock and upper arm. In order to avoid infections, beyond the chosen site, an appropriate cleaning of the area is necessary before administration [3]. After adequately priming the device, the recommended 4-mm pen needle should be inserted at 90° in the skin so that insulin is safely released into subcutaneous fat tissue and the thumb button should be kept pressed until the patient slowly count to 10 [3]. Most frequent skin-related adverse event associated with IT is lipohypertrophy due to the lack of skin spacing and rotation of injection site [3, 4]. Recommendations suggest that the injection site should be changed at least every four weeks and a correct spacing consists of 1 fingerbreadth (approximately 1 cm) [3]. The risk of intramuscular injection is another challenge, both related to patient characteristic such as skin and subcutaneous thickness and needle length. Intramuscular administration of insulin leads to poor glycemic control and higher risk of hypoglycemia because of the extreme variability in insulin absorption through muscles (especially into exercising muscles) [3]. A further well-known problem is the failure to remove the inner needle cap that makes the patient not taking the insulin dose and, consequently, glycemic control is not achieved [5]. Our clinical case highlights the possibility of other and various adverse events related both to misuse of insulin devices and to the presence of disabling complication. Specifically, our patient was treated with insulin for two years and received a sufficient education on the correct use of the device, but his severe peripheral neuropathy compromised his ability to safely remove the needle. This clinical case underlines that knowledge, skills of patients and their education and counseling in the correct use of insulin pens as well as the regular check of their handling are of crucial importance and strictly recommended to ensure effectiveness and safety of treatments. Moreover, clinicians should be aware that comorbidities and complications of the disease may impact on compliance to devices and their correct use.

## Data Availability

Not applicable.
